# Revisiting Tregs in cancer and beyond: immunological control and therapeutic potential

**DOI:** 10.3389/fimmu.2025.1581093

**Published:** 2025-09-01

**Authors:** Lei Zheng, Dan Wu, Hongwei Xie, Hai Zhao

**Affiliations:** ^1^ Department of Emergency Surgery, the Affiliated Hospital of Qingdao University, Qingdao, Shandong, China; ^2^ Department of Neurosurgery, the Affiliated Hospital of Qingdao University, Qingdao, Shandong, China

**Keywords:** regulatory T cells, immune suppression, cancer immunotherapy, tissue repair and regeneration, metabolic regulation, angiogenesis

## Abstract

Tregs play a crucial role in maintaining immune homeostasis, but their involvement in cancer and other diseases has made them a focus of intense research. Tregs contribute to immune evasion by tumors and can affect responses to therapies. Understanding their mechanisms and the potential to manipulate them therapeutically is critical for improving cancer treatment strategies. This review aims to provide an updated perspective on the role of Tregs in cancer and beyond, with a focus on their immunological control mechanisms and therapeutic potential. We examine the recent advances in understanding Treg biology, their interaction with the tumor microenvironment, and the strategies developed to target Tregs for cancer immunotherapy. The review highlights the dual role of Tregs in promoting immune tolerance and in facilitating tumor progression. It discusses the various markers, transcription factors, and signaling pathways involved in Treg differentiation and function. Moreover, we explore the potential of targeting Tregs using novel therapeutic approaches, including monoclonal antibodies, checkpoint inhibitors, and gene editing. The review emphasizes emerging strategies for modulating Treg function in a way that enhances anti-tumor immunity while minimizing systemic autoimmunity.

## Introduction

Regulatory T cells (Tregs) are a specialized subset of T cells essential for maintaining immune homeostasis and preventing autoimmune responses. These cells are characterized by the expression of the transcription factor Foxp3 (Forkhead box P3), which is crucial for their development and function (1). Tregs originate from precursor cells in the thymus and are marked by high levels of the interleukin-2 receptor alpha chain (CD25) and CD4 expression (2). Their primary function is to suppress aberrant immune responses that could lead to autoimmunity, thereby maintaining the delicate balance of the immune system. Tregs can be broadly classified into natural Tregs (nTregs), which develop in the thymus, and induced Tregs (iTregs), which differentiate from conventional CD4+ T cells in the periphery under specific conditions, such as the presence of transforming growth factor-beta (TGF-β) and IL-2 (3).

The role of Tregs in cancer is complex, involving both tumor-promoting and tumor-suppressing activities (4). In the tumor microenvironment, Tregs often accumulate and suppress anti-tumor immune responses, aiding in tumor survival and progression (5). This immunosuppressive environment enables tumor cells to evade immune surveillance. Recent therapeutic advancements have focused on modulating Treg activity to enhance cancer immunotherapy (5–7). Strategies include selectively depleting Tregs within tumors using agents targeting CD25 or cytotoxic T-lymphocyte associated protein 4 (CTLA-4), and engineering Tregs with chimeric antigen receptors (CARs) to improve their specificity and function against tumors (8–12). These approaches aim to disrupt the immunosuppressive shield around tumors while preserving the overall immune balance, thereby enhancing the efficacy of immune checkpoint inhibitors and other cancer therapies.

Beyond their immunosuppressive roles, Tregs exhibit several non-canonical functions that are critical for various physiological processes. Tregs play a significant role in tissue repair and regeneration by secreting cytokines and growth factors like amphiregulin, which aid in wound healing and the restoration of tissue integrity (13, 14). In metabolic regulation, Tregs influence lipid metabolism and insulin sensitivity, particularly in adipose tissues where they help maintain metabolic homeostasis (15, 16). Tregs are also crucial in supporting stem cell niches, such as those in the bone marrow, skin, and intestines, where they maintain stem cell quiescence and function through direct interactions and cytokine secretion (17). Additionally, Tregs regulate angiogenesis, the formation of new blood vessels, by balancing pro- and anti-angiogenic factors, which is also essential for both tissue repair and tumor progression (18).

This review aims to provide a comprehensive overview of the diverse roles of Tregs in immune regulation, cancer therapy, and beyond. By exploring the multifaceted functions of Tregs, we seek to highlight their potential as therapeutic targets in various diseases, including cancer, autoimmune disorders, and conditions like tissue regeneration. Understanding the complex interplay between Tregs and their environment is crucial for developing innovative therapies that can modulate these cells to achieve desired clinical outcomes. This synthesis of current knowledge underscores the importance of Tregs in both health and disease, offering insights into future research directions and therapeutic strategies.

## Treg origin and identity

Tregs are a specialized subset of T cells that play a crucial role in maintaining immune homeostasis and preventing excessive immune responses. Like other T cell subsets, Tregs develop in the thymus from precursor cells. They undergo specific differentiation processes, including the expression of the transcription factor Foxp3, which is considered a master regulator of Treg development and function (19–21). Tregs are characterized by the expression of certain surface markers, of which the most used marker for identifying is CD4. Additionally, Tregs express high levels of CD25 and the transcription factor Foxp3, which is considered a key marker of Treg identity.

Current research revealed that the gut microbiota is pivotal in shaping immune development early in life. *Sanidad et al.* revealed that the microbiota in the small intestine of neonatal mice significantly differs from that in adults, notably enriched with neurotransmitters such as serotonin (22). Both human and mouse neonates exhibit a higher concentration of bacteria that produce 5-HT (5-Hydroxytryptamine) in their small intestines. In neonates, gut bacteria not only boost 5-HT production by activating the Tryptophan Hydroxylase 1 enzyme, which transforms tryptophan into 5-HT, but also enhance its availability by suppressing MAO-A (Monoami ne Oxidase A), an enzyme that breaks down 5-HT (22). This increase in serotonin uniquely influences T cell metabolism in neonates by raising the levels of indole-3-acetaldehyde (I3A), which suppresses the mTOR pathway and facilitates the development of Tregs (22, 23). Administering 5-HT orally to neonatal mice, but not to adults, fosters systemic and enduring immune tolerance to dietary antigens and commensal bacteria, illustrating a mechanism through which gut bacteria promote oral tolerance in early life (22).

In our discussion on Tregs, we now explicitly differentiate between nTregs and iTregs. nTregs are characterized by their development in the thymus as a distinct lineage of T cells, where the expression of the transcription factor Foxp3 plays a pivotal role in their differentiation and function. nTregs are essential for maintaining self-tolerance and immune homeostasis, preventing autoimmune diseases by suppressing aberrant immune responses against self-antigens (3).

Conversely, iTregs are generated from conventional CD4+ T cells in the periphery under certain conditions, notably in the presence of TGF-β and IL-2. iTregs also express Foxp3 and share suppressive functions like nTregs; however, their induction, stability, and roles in immune responses, especially in the context of inflammation and tolerance to non-self-antigens, distinguish them from nTregs. The plasticity of iTregs and their ability to arise from effector T cells under specific microenvironmental cues underscore their versatility in immune regulation (24).

By elaborating on these differences, we aim to provide a comprehensive understanding of the diverse regulatory mechanisms mediated by these two Treg subsets, further enriching the discussion on their significance in immune tolerance and potential therapeutic implications.

## Canonical mechanisms of immune regulation by Tregs

**Figure 1:**
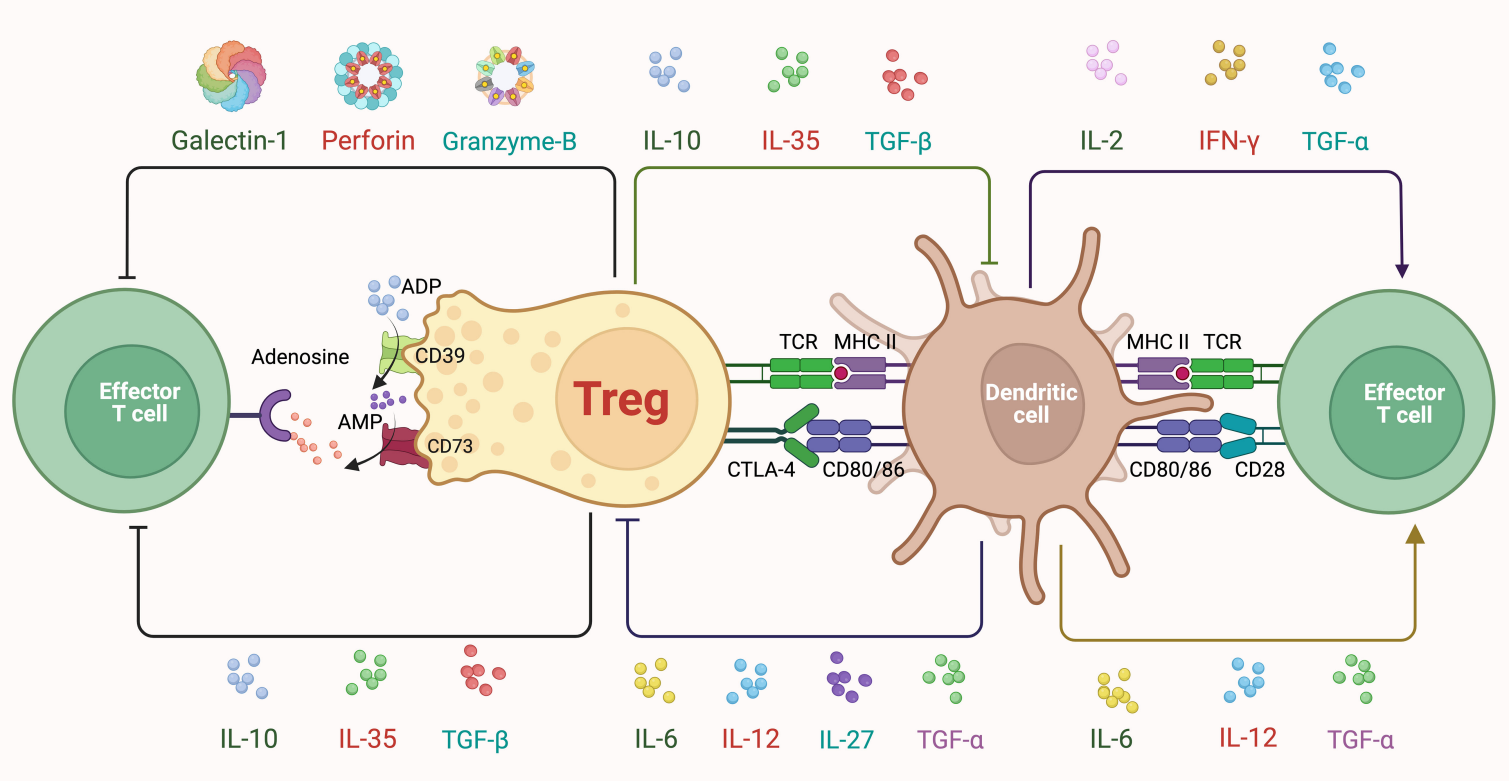
Classic mechanisms of treg suppression. Tregs express high levels of CD25, allowing them to efficiently capture and consume IL-2. This deprives nearby effector T cells of IL-2, limiting their proliferation and activity, and thereby contributing to immune suppression. Moreover, Tregs secrete several cytokines, including IL-10, IL-35, and TGF-β, which inhibit the activation and function of both DCs and effector T cells. IL-10 reduces the expression of co-stimulatory molecules and proinflammatory cytokines while IL-35 suppresses effector T cell proliferation, and TGF-β inhibits effector T cell activation while promoting the differentiation of other Tregs. Metabolically, Tregs express CD39 and CD73, converting ATP (Adenosine Triphosphate) and ADP (Adenosine Diphosphate) to adenosine, which then binds to adenosine receptors on effector T cells, leading to their suppression. Additionally, Tregs secrete Galectin-1, which induces apoptosis in effector T cells. Tregs also utilize cell-cell contact-dependent mechanisms such as CTLA-4, which competes with CD28 on effector T cells for binding to CD80/CD86 on DCs, thereby reducing co-stimulatory signals necessary for effector T cell activation. Tregs interact directly with DCs through TCR (T Cell Receptor) and MHC II (major histocompatibility complex class II) interactions, modulating DC function and indirectly suppressing effector T cell activation. Furthermore, Tregs can induce apoptosis in effector T cells via the release of cytotoxic molecules like perforin and granzyme B.

Tregs exert their immunosuppressive and regulatory functions through various mechanisms (**Figure 1**): i. Consumption of IL-2: One of the central mechanisms by which Tregs exert their immunosuppressive function is through the high-affinity CD25, which enables them to outcompete conventional T cells for IL-2 (3). By consuming IL-2 in a CD25-dependent manner, Tregs effectively deprive the surrounding effector T cell populations of this critical growth factor, thereby limiting their expansion and activity. This IL-2 consumption mechanism represents a non-cytolytic, contact-independent strategy that is particularly relevant in maintaining peripheral tolerance and preventing excessive immune activation. ii. Direct Cell-to-Cell Suppression: Tregs can directly interact with other immune cells, such as effector T cells, DCs (dendritic cells), and B cells, through cell-to-cell contact. This interaction leads to the suppression of immune responses and the inhibition of the activation and proliferation of effector T cells (25). iii. Secretion of Suppressive Molecules: Treg cells exhibit the production of various cytokines, including IL-10, TGF-β, IL-35 and growth differentiation factor 15 (GDF15), which play pivotal roles in immune regulation and tolerance maintenance (26, 27). These cytokines contribute to the suppression of effector T cell responses and the promotion of immune homeostasis, highlighting the multifaceted regulatory functions of Treg cells in modulating immune responses. iv. Metabolic Regulation: Tregs can modulate the metabolism of effector T cells and alter their nutrient availability, thereby suppressing their proliferation and effector functions (28).

## Classic mechanisms of Treg suppression

Tregs express high levels of CD25, allowing them to efficiently capture and consume IL-2. This deprives nearby effector T cells of IL-2, limiting their proliferation and activity, and thereby contributing to immune suppression (29, 30). Moreover, Tregs secrete several cytokines, including IL-10, IL-35, and TGF-β, which inhibit the activation and function of both DCs and effector T cells. IL-10 reduces the expression of co-stimulatory molecules and pro-inflammatory cytokines while IL-35 suppresses effector T cell proliferation, and TGF-β inhibits effector T cell activation while promoting the differentiation of other Tregs (31). Metabolically, Tregs express CD39 and CD73, converting ATP (Adenosine Triphosphate) and ADP (Adenosine Diphosphate) to adenosine, which then binds to adenosine receptors on effector T cells, leading to their suppression (2, 32). Additionally, Tregs secrete Galectin-1, which induces apoptosis in effector T cells (33). Tregs also utilize cell-cell contact-dependent mechanisms such as CTLA-4, which competes with CD28 on effector T cells for binding to CD80/CD86 on DCs, thereby reducing co-stimulatory signals necessary for effector T cell activation (34). Tregs interact directly with DCs through TCR (T Cell Receptor) and MHC II (major histocompatibility complex class II) interactions, modulating DC function and indirectly suppressing effector T cell activation. Furthermore, Tregs can induce apoptosis in effector T cells via the release of cytotoxic molecules like perforin and granzyme B (35).

### Novel immune regulation mechanisms of Tregs

The immune regulatory mechanisms of Tregs exhibit high complexity, involving multi-layered interactions at the molecular, metabolic, and microenvironmental levels. These interactions are pivotal for maintaining immune homeostasis and for the modulation of immune responses within various contexts, including cancer. In tumor microenvironment, Tregs play a dual role by not only maintaining immune tolerance but also potentially facilitating tumor escape from immune surveillance (36–38). This paradoxical nature makes understanding the novel immune regulatory mechanisms of Tregs particularly significant, as it opens potential therapeutic avenues for tipping the balance in favor of tumor suppression without compromising systemic immune tolerance.

Firstly, metabolic adaptation plays a crucial role, as Tregs optimize their metabolism to survive in the nutrient-sparse tumor microenvironment by primarily using fatty acid oxidation (39). This enables them to maintain suppressive functionality under challenging conditions. Secondly, Tregs utilize exosome-mediated communication to extend their influence within the tumor microenvironment (40). These exosomes, loaded with microRNAs and other regulatory molecules, modulate the behavior of other immune cells and contribute to tumor immune escape (41).

Additionally, exploration of novel immune checkpoints such as TIGIT (T-cell Immunoreceptor with Ig and ITIM Domains) and LAG-3 (Lymphocyte-activation gene 3) extends beyond classic pathways like CTLA-4 and PD-1 (programmed cell death protein 1), providing new avenues for immunotherapeutic targets that influence Treg activity (42–44). Moreover, the plasticity of Tregs is highlighted by their potential conversion into ex-Tregs under specific environmental cues, which can then switch from suppressing to promoting immune responses. This plasticity underlines the dynamic nature of Tregs in response to the tumor microenvironment.

Furthermore, the role of hypoxia-inducible factors such as HIF1-α (Hypoxia-Inducible Factor 1-Alpha) is critical in adapting Treg functions to low-oxygen conditions typical in solid tumors (45, 46). HIF1-α enhances the stability and functionality of FoxP3, promoting Treg survival and suppressive activity under hypoxia (47). By elucidating these mechanisms, the manuscript can provide a detailed perspective on how Tregs crucially modulate the tumor microenvironment, reflecting the intricacy of their roles and the potential for targeted therapies that manipulate their regulatory functions. This comprehensive analysis will not only align with the latest research trends but also deepen our understanding of Treg dynamics within cancer biology, offering insights into potential therapeutic interventions.

The novel immune regulatory mechanisms of Tregs, especially within the context of the tumor microenvironment, underscore a critical area of oncological research that bridges immunology and tumor biology. The dynamic interplay between Tregs and the tumor microenvironment highlights the crucial role these cells play in modulating immune responses directly at the tumor site. Tregs not only influence the recruitment and activation of other immune cells through their secretory and cell-contact dependent mechanisms but also adapt to the harsh, often hypoxic and metabolically restricted conditions of the tumor microenvironment. This adaptation supports their survival and functional persistence, which in turn can either promote or inhibit tumor growth depending on the context.

Understanding these complex interactions is vital for developing targeted therapies that can modulate Treg activity to enhance anti-tumor immunity without inducing systemic autoimmunity. The insights gained from studying Treg interactions in the tumor microenvironment could lead to the development of biomarkers for better prognosis and tailored therapeutic strategies that specifically target the immune regulation in the tumor niche. This could potentially transform the approach to cancer therapy, moving towards more personalized and effective immunomodulatory treatments.

### Recent advancements in Treg diversity

Recent studies have revealed that Tregs are not a homogenous population but rather comprise distinct subsets with specialized functions. These subsets, such as tissue-resident Tregs and follicular Tregs, have unique phenotypes and exhibit specific regulatory roles in different tissues and immune contexts (48–57). Traditionally, Tregs were considered stable and committed to their suppressive function. However, recent research has highlighted that Tregs can exhibit plasticity and acquire effector-like characteristics under certain conditions (58–62). This plasticity raises important questions about the stability of Treg function and their potential roles in autoimmune diseases and cancer. The tissue microenvironment plays a crucial role in shaping the functions and stability of Tregs (63, 64). Recent studies have focused on understanding the signals and cues within specific tissues that influence Treg function and stability (51, 65–67). This knowledge can provide insights into how Tregs adapt their regulatory properties based on the local tissue environment. In addition, Tregs have garnered significant attention as potential therapeutic agents for immune-mediated diseases (68, 69). Advances in the isolation, expansion, and genetic engineering of Tregs have opened up possibilities for their use in immunotherapy, including in the context of transplantation, autoimmune diseases, and graft-versus-host disease (70–72). Recent advances have deepened our understanding of Tregs and their roles in immune regulation. They hold promise for developing targeted strategies to modulate immune responses, restore immune balance, and potentially treat various immune-related disorders.

One fascinating area of progress is the development of antigen-specific regulatory T cells (73, 74). These cells have shown higher potency in immuno-suppression compared to polyclonal T cells in preclinical mouse models. Recent technical advances aim to generate antigen-specific Treg cells in sufficient quantities for therapeutic use, which includes redirecting Tregs using synthetic receptors and converting antigen-specific effector T cells into Tregs through FOXP3 overexpression. The use of CAR constructs is prevalent due to their ability to recognize a wide range of antigens, including non-protein targets. These advances have shown promise in treating hematological malignancies and offer a new strategy for autoimmune disease therapy and transplantation ​ (75, 76).

The present research identified a distinct population of meningeal regulatory T cells (mTregs) residing in the meninges of the spinal cord and the leptomeninges surrounding dorsal root ganglia (77). These mTregs were enriched in the lumbosacral meninges and were found in proximity to IB4⁺ non-peptidergic sensory nerve fibers, indicating potential neuroimmune interaction (78). Using Foxp3-DTR mice, site-specific depletion of mTregs via intrathecal injection of PEGylated diphtheria toxin (pegDT) led to a sex-specific effect: only female mice exhibited reduced mechanical nociceptive thresholds, suggesting that mTregs exert a hormone-dependent analgesic function (77, 79). Conversely, intrathecal administration of low-dose IL-2 after nerve injury selectively expanded mTregs and alleviated mechanical hypersensitivity in females (77). These findings establish mTregs as key regulators of nociception in a sex-dependent manner and suggest that they may act independently of classical immune-suppressive pathways. The results provide novel insight into neuroimmune mechanisms of pain regulation and may help explain sex differences in pain sensitivity, offering potential for gender-specific pain therapies (77).

### Tregs in cancer: a dual role

**Figure 2:**
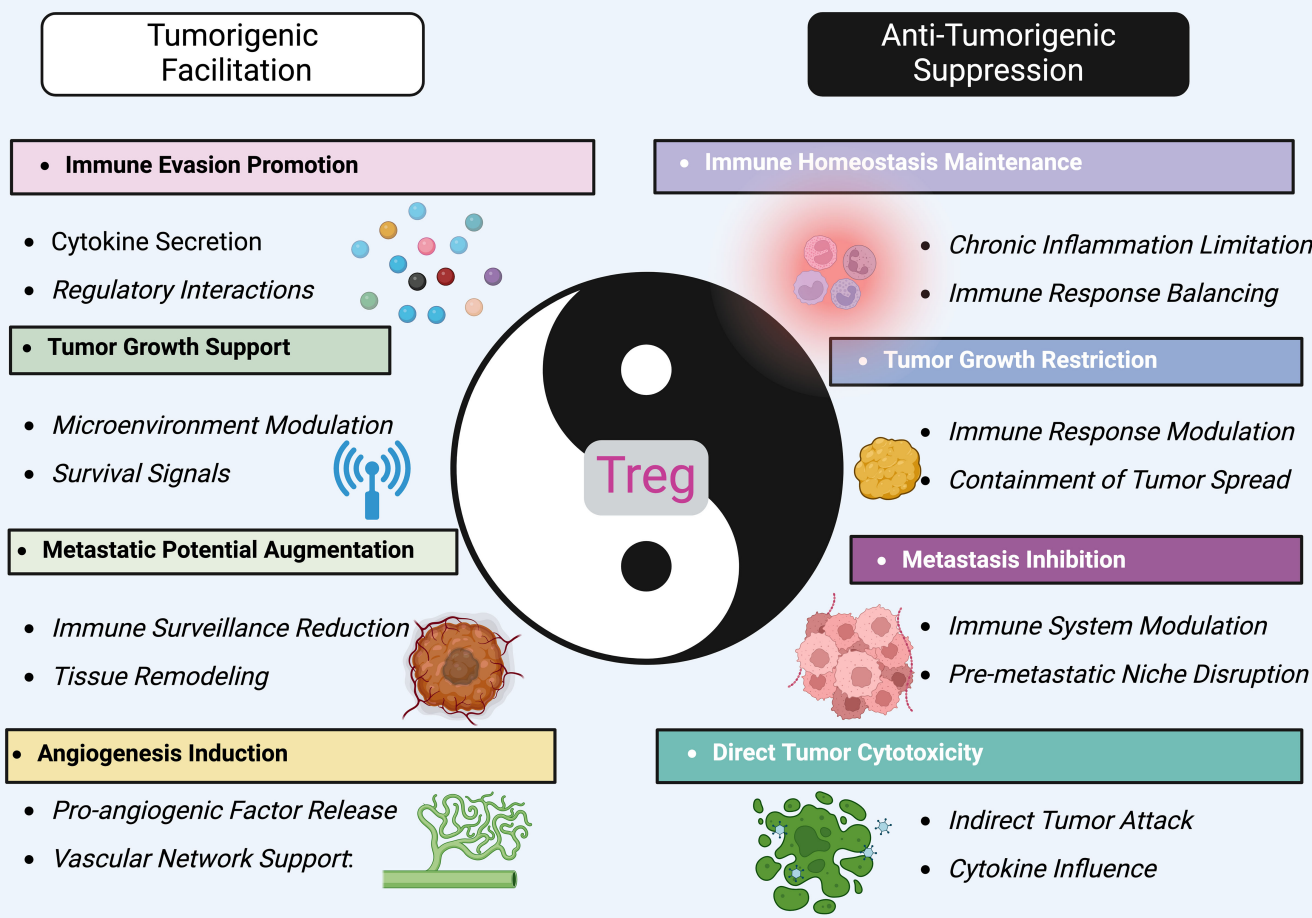
The dual role of treg in cancer. Tregs embody a paradigm of immunological dualism in the tumor microenvironment, oscillating between tumorigenesis facilitation and antitumor activity. On one side, Tregs promote tumorigenic processes, such as immune evasion, by secreting cytokines that modify the immune landscape to favor tumor growth and survival. They support the tumor by enhancing angiogenesis, which supplies necessary nutrients and oxygen, and by modulating the microenvironment to suppress anti-tumor immune surveillance. This leads to augmented metastatic potential and survival signaling within the tumor. Conversely, Tregs also exert anti-tumorigenic effects. They help maintain immune homeostasis, limiting chronic inflammation and immune responses that could otherwise support tumor progression. Tregs can restrict tumor growth by modulating immune responses that contain and potentially reduce tumor spread. Their role in inhibiting metastasis involves disrupting the formation of pre-metastatic niches, crucial for preventing the establishment of secondary tumors. Furthermore, Tregs can directly impact tumor cells through cytotoxic activities, though less commonly. They might indirectly attack tumor cells or influence other immune cells to target the tumor, mediated by their control over cytokine environments. By targeting specific pathways that influence Treg activity, it might be possible to shift their balance towards more consistent anti-tumorigenic actions, offering new avenues for therapeutic interventions in cancer management.

Tregs play a complex role in cancer, and their involvement can have both positive and negative effects on tumor progression (**Figure 2**): i. One of the primary functions of Tregs is to suppress immune responses, including those targeting tumor cells. Tregs can inhibit the activation and effector functions of cytotoxic T cells and others, thereby dampening anti-tumor immune responses. This immunosuppressive effect can create an environment that allows tumor cells to evade immune surveillance and promote tumor growth. ii. Tregs also play a crucial role in maintaining immune tolerance and preventing excessive immune reactions against self-antigens. In the context of cancer, this can result in Tregs suppressing immune responses against tumor-associated antigens, which are derived from normal self-proteins that are aberrantly expressed by tumor cells. iii. Tregs have been found to infiltrate tumors and are often enriched within the tumor microenvironment (80–82). The presence of Tregs within tumors has been associated with poor clinical outcomes in some cancers (83, 84). Tumor-infiltrating Tregs can actively suppress anti-tumor immune responses, promote immune evasion, and contribute to tumor immune evasion and progression. iv. Tregs can also contribute to resistance to certain cancer immunotherapies, such as immune checkpoint blockade (ICB) therapy. Tregs can upregulate immune checkpoint molecules, such as CTLA-4 and PD-1, which can dampen the activity of anti-tumor immune cells and limit the efficacy of ICB therapy (85). Targeting Tregs alongside ICB therapy is an active area of research to enhance treatment responses (86, 87). v. Some evidence suggests that Tregs can acquire tumor-specific antigen reactivity, leading to their differentiation into tumor-associated Tregs (TATs) (88–91). TATs exhibit an enhanced ability to suppress anti-tumor immune responses, further promoting tumor immune evasion (90, 92, 93). vi. Despite their suppressive role in anti-tumor immunity, Tregs are also involved in maintaining tissue homeostasis and preventing chronic inflammation, which can contribute to tumorigenesis (94, 95). Additionally, recent research suggests that not all Tregs exhibit the same suppressive properties, and subsets with pro-inflammatory properties or compromised suppressive function have been identified in certain cancer contexts (58–62). Targeting Tregs as a therapeutic strategy in cancer is an active area of investigation (96, 97). Several approaches are being explored, such as selectively depleting or inhibiting Tregs within tumors or modulating their function to enhance anti-tumor immune responses (62, 98). Combining Treg-targeted therapies with other immunotherapeutic approaches holds promise for improving outcomes in cancer treatment.

As research on Tregs evolves, the earlier notion that Tregs uniformly result in unfavorable cancer outcomes is increasingly recognized as oversimplified. Indeed, Tregs exhibit a dual role in the progression and onset of tumors. They mitigate systemic inflammation caused by overly active anti-tumor immune responses, crucial for maintaining immune equilibrium. Conversely, in advanced tumor stages, Tregs may attenuate these anti-tumor responses, promoting tumor cell survival. At earlier stages, their anti-inflammatory actions can inadvertently favor tumor growth. For instance, RORγT+ Helios− Tregs, activated by intestinal microbes, respond to IL-33 triggered by tissue damage, helping limit injury and forestall cancer development during colitis (99). In head and neck cancers, the accumulation of Foxp3+ Tregs correlates with improved prognoses, likely reflecting their diverse functions within different TMEs (tumor microenvironment) affecting their activity in various tumor areas (100, 101). In colorectal cancer, Tregs found in the tumor mesenchyme are associated with better outcomes, whereas those in tumor nests or edges correlate with worse prognoses (102, 103). Moreover, the impact of Tregs varies across cancer stages, with high Foxp3+ Treg presence in early gastric cancer stages linked to better survival rates, a trend that inverses in later stages. These discrepancies suggest changes in Treg function depending on the TME, influencing their effects on tumor prognosis and treatment strategies. Although it is speculative, the dual role of Tregs in tumor biology demands further investigation to elucidate their complex behaviors fully, advancing our understanding and application of Tregs in cancer therapeutics (**Figure 2**).

### The dual role of treg in cancer

Tregs embody a paradigm of immunological dualism in the tumor microenvironment, oscillating between tumorigenesis facilitation and antitumor activity. On one side, Tregs promote tumorigenic processes, such as immune evasion, by secreting cytokines that modify the immune landscape to favor tumor growth and survival. They support the tumor by enhancing angiogenesis, which supplies necessary nutrients and oxygen, and by modulating the microenvironment to suppress anti-tumor immune surveillance. This leads to augmented metastatic potential and survival signaling within the tumor (104, 105). Conversely, Tregs also exert anti-tumorigenic effects. They help maintain immune homeostasis, limiting chronic inflammation and immune responses that could otherwise support tumor progression (106). Tregs can restrict tumor growth by modulating immune responses that contain and potentially reduce tumor spread (107–109). Their role in inhibiting metastasis involves disrupting the formation of pre-metastatic niches, crucial for preventing the establishment of secondary tumors (110, 111). Furthermore, Tregs can directly impact tumor cells through cytotoxic activities, though less commonly (112, 113). They might indirectly attack tumor cells or influence other immune cells to target the tumor, mediated by their control over cytokine environments. By targeting specific pathways that influence Treg activity, it might be possible to shift their balance towards more consistent anti-tumorigenic actions, offering new avenues for therapeutic interventions in cancer management (114).

### Treg functions beyond immune suppression

**Figure 3:**
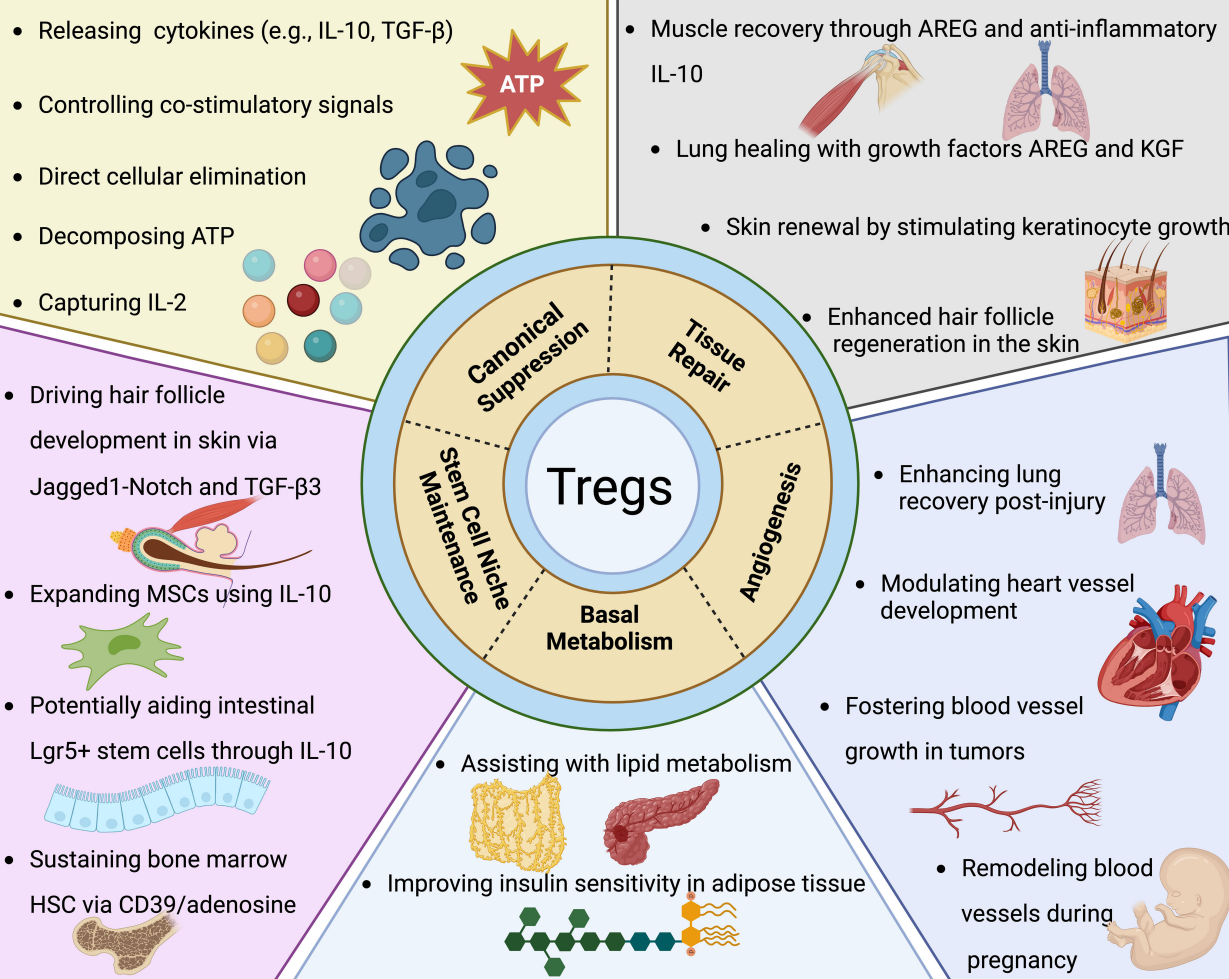
Tregs extend their functional repertoire well beyond mere immune suppression. As elaborated in the segment concerning canonical suppression, Tregs employ multiple strategies to curtail the proliferation and operational capacity of effector T cells alongside various other immune constituents. Moreover, Tregs exert extensive influence over processes such as tissue regeneration, angiogenesis, fundamental metabolism, and the preservation of stem cell microenvironments. Their contributions to tissue repair have been predominantly observed in muscle, lung, skin, and the central nervous system. Key to these processes are Treg-derived factors like Areg (amphiregulin) and Keratinocyte growth factor, which are essential for the proliferative responses of epithelial and satellite cells in lung, skin, and muscle tissues, respectively. Within the CNS (central nervous system), Tregs bolster the capacity of oligodendrocytes to restore myelin sheaths post-injury. The angiogenic role of Tregs is context-dependent, exhibiting both pro-angiogenic and anti-angiogenic activities based on the tissue type and experimental model. In the realm of oncology, Tregs distinctly support tumor angiogenesis, facilitating neoplastic growth. Their involvement in ischemic conditions reveals a complex picture: Tregs are necessary for revascularization in lung injury yet may counteract angiogenesis in cardiac ischemia. They also partake in the vascular adjustments occurring within the uterus throughout pregnancy. Specifically, visceral adipose tissue Tregs, which are among the most thoroughly characterized tissue-resident Tregs, play definitive roles in modulating insulin sensitivity and lipid metabolism. Additionally, Tregs underpin the specialized stem cell niches within the skin, bone marrow, and gastrointestinal tract. Through a variety of mechanisms, they directly uphold stem cell dormancy and indirectly modulate these cells by limiting the proliferation of the supporting mesenchymal stromal cells. While several of these observations have been corroborated in human studies, it's critical to acknowledge that a substantial portion of the insights is derived from murine research.

In addition to their well-characterized roles in maintaining immune homeostasis and preventing autoimmunity, Tregs exhibit a range of other regulatory functions that are crucial for their versatility in the immune system. These expanded functions include modulation of tissue repair, influence on metabolic processes, and participation in the resolution of inflammation, highlighting the multifaceted roles Tregs play beyond traditional immune suppression (**Figure 3**).

### Tregs extend their functional repertoire well beyond mere immune suppression

As elaborated in the segment concerning canonical suppression, Tregs employ multiple strategies to curtail the proliferation and operational capacity of effector T cells alongside various other immune constituents. Moreover, Tregs exert extensive influence over processes such as tissue regeneration, angiogenesis, fundamental metabolism, and the preservation of stem cell microenvironments. Their contributions to tissue repair have been predominantly observed in muscle, lung, skin, and the central nervous system. Key to these processes are Treg-derived factors like Areg (amphiregulin) and Keratinocyte growth factor, which are essential for the proliferative responses of epithelial and satellite cells in lung, skin, and muscle tissues, respectively. Within the CNS (central nervous system), Tregs bolster the capacity of oligodendrocytes to restore myelin sheaths post-injury. The angiogenic role of Tregs is context-dependent, exhibiting both pro-angiogenic and anti-angiogenic activities based on the tissue type and experimental model. In the realm of oncology, Tregs distinctly support tumor angiogenesis, facilitating neoplastic growth. Their involvement in ischemic conditions reveals a complex picture: Tregs are necessary for revascularization in lung injury yet may counteract angiogenesis in cardiac ischemia. They also partake in the vascular adjustments occurring within the uterus throughout pregnancy. Specifically, visceral adipose tissue Tregs, which are among the most thoroughly characterized tissue-resident Tregs, play definitive roles in modulating insulin sensitivity and lipid metabolism. Additionally, Tregs underpin the specialized stem cell niches within the skin, bone marrow, and gastrointestinal tract. Through a variety of mechanisms, they directly uphold stem cell dormancy and indirectly modulate these cells by limiting the proliferation of the supporting mesenchymal stromal cells. While several of these observations have been corroborated in human studies, it’s critical to acknowledge that a substantial portion of the insights is derived from murine research.Tissue repair and regeneration

**Figure 4:**
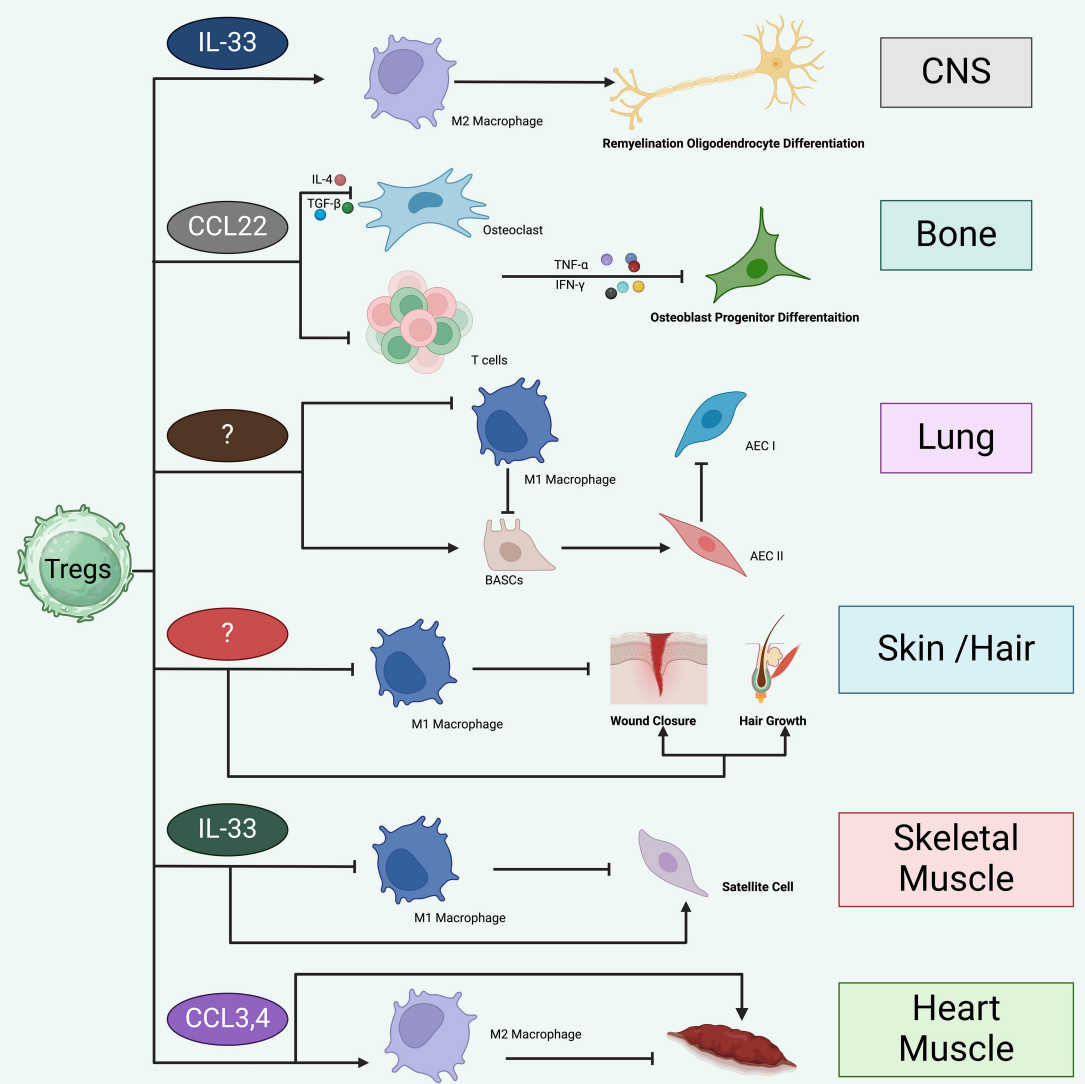
Treg likely promote tissue repair and regeneration in a tissue-specific manner. Tregs appear to facilitate tissue repair and regeneration in a manner specific to each tissue type. Tregs are crucial in the healing and regeneration processes of various tissues, including skeletal muscle, cardiac muscle, skin and hair, lungs, bones, and CNS. i. In the CNS, Tregs are drawn by IL-33 and contribute to repair by promoting M2 macrophage polarization, which aids in remyelination and the differentiation of oligodendrocytes. Additionally, Tregs may directly influence oligodendrocytes through mechanisms involving CCN3. ii. In bone tissue, Tregs are likely recruited through CCL22 signaling and function to inhibit Th1, CD8+ cells, and M1 macrophages, thereby promoting differentiation of osteoblast progenitors. iii. In the lung, Tregs suppress the inflammatory responses of M1 macrophages and support the proliferation and differentiation of damaged alveolar type 2 epithelial cells (AECII) into type 1 cells (AECIs). This activity may be mediated by Areg or the interaction of CD103 with E-cadherin. Tregs may also activate progenitor bronchioalveolar stem cells (BASCs) to differentiate into AECII cells while concurrently preventing fibrosis through the inhibition of fibrocyte recruitment and proliferation, mediated by CXCL12. iv. The mechanism by which Tregs are recruited to skin and hair remains unclear. However, once present, Tregs curb the inflammatory actions of M1 macrophages and enhance wound healing and hair regeneration through the Jag1-Notch signaling pathway. v. Within skeletal muscle, IL-33 aids in the recruitment of Tregs to injury sites. These cells suppress inflammation mediated by M1 macrophages, thereby fostering a shift towards the resolution phase of healing. Tregs also directly stimulate satellite cell proliferation and differentiation through Areg. vi. In cardiac tissue, Tregs are drawn to the site via CCR5 signaling, involving chemokines like CCL3 and CCL4, which leads to the suppression of Th1 cell activities and inhibition of M1 macrophages.

Recent advancements have expanded the understanding of Treg function, revealing significant contributions beyond traditional immunoregulation, particularly in the realms of tissue repair and regeneration. Tregs likely promote tissue repair and regeneration in a tissue-specific manner by modulating local immune responses and supporting tissue-specific progenitor cells. In the CNS, Tregs facilitate remyelination and oligodendrocyte differentiation via IL-33 and CCN3 signaling. In the lung, they promote alveolar epithelial cell regeneration and limit fibrosis. In bone, they suppress pro-inflammatory cells and enhance osteoblast differentiation. In skeletal muscle and cardiac tissue, Tregs are recruited through IL-33 and CCR5 (C-C chemokine receptor type 5) pathways, respectively, to modulate macrophage activity and support tissue regeneration. These findings highlight the context-dependent reparative roles of Tregs across organs (**Figure 4**). In tissue repair and regeneration, Tregs facilitate wound healing and reduce chronic inflammation that can interfere with the recovery of damaged tissues (115, 116). This function is evident across various tissue types, including skin, muscle, and pulmonary systems, where Tregs have been observed to enhance wound closure rates and improve structural restoration post-injury (116).

The molecular mechanisms underlying Treg involvement in tissue repair involve a sophisticated network of cytokines and growth factors. Tregs are known to secrete anti-inflammatory cytokines such as IL-10 and TGF-β (116). IL-10 contributes to the suppression of effector T cell activities and decreases the production of pro-inflammatory cytokines, thereby promoting an environment conducive to healing (117). TGF-β, on the other hand, supports fibrosis and angiogenesis, essential processes in tissue repair (118–120). Interactions between Tregs and other cell types within the damaged tissue are crucial. Tregs influence macrophages to adopt a reparative M2 phenotype, characterized by the secretion of growth factors and enzymes that aid in matrix remodeling and cellular proliferation (121, 122). A specific role of Tregs in regenerative processes includes their expression of Areg, a member of the epidermal growth factor family (123). Areg promotes the proliferation of epithelial cells, critical for restoring barrier functions in tissues such as the skin and gastrointestinal tract (123).

Tissue-resident ST2⁺ Tregs represent a specialized subset with pivotal roles in tissue repair and homeostasis (124). These cells are characterized by the expression of the IL-33 receptor ST2 and are enriched in non-lymphoid tissues such as the lung, muscle, and adipose tissue, particularly under conditions of injury or inflammation (124). Upon sensing IL-33, an alarmin released by damaged epithelial or stromal cells, ST2⁺ Tregs become activated and secrete Areg, which promotes epithelial proliferation and tissue regeneration (125).

However, the molecular pathways driving tissue repair, including IL-33/ST2 and Areg/EGFR signaling, are frequently co-opted by tumors to facilitate malignant progression (126). ST2⁺ Tregs have been reported to accumulate in various tumor microenvironments, where they may contribute not only to immune suppression but also to tumor-promoting inflammation and stromal remodeling via the same regenerative programs (127). The overlap between regenerative and tumorigenic signaling suggests that ST2⁺ Tregs play a dual role—beneficial in resolving inflammation and restoring tissue integrity, yet potentially deleterious in promoting tumor growth and metastasis (127). Therefore, a comprehensive understanding of their context-dependent functions is essential for designing targeted therapies that harness their reparative potential without exacerbating cancer progression.

### Treg likely promote tissue repair and regeneration in a tissue-specific manner

Tregs appear to facilitate tissue repair and regeneration in a manner specific to each tissue type. Tregs are crucial in the healing and regeneration processes of various tissues, including skeletal muscle, cardiac muscle, skin and hair, lungs, bones, and CNS. i. In the CNS, Tregs are drawn by IL-33 and contribute to repair by promoting M2 macrophage polarization, which aids in remyelination and the differentiation of oligodendrocytes (128, 129). Additionally, Tregs may directly influence oligodendrocytes through mechanisms involving CCN3 (130). ii. In bone tissue, Tregs are likely recruited through CCL22 signaling and function to inhibit Th1, CD8+ cells, and M1 macrophages, thereby promoting differentiation of osteoblast progenitors (131). iii. In the lung, Tregs suppress the inflammatory responses of M1 macrophages and support the proliferation and differentiation of damaged alveolar type 2 epithelial cells (AECII) into type 1 cells (AECIs) (132). This activity may be mediated by Areg or the interaction of CD103 with E-cadherin (133). Tregs may also activate progenitor bronchioalveolar stem cells (BASCs) to differentiate into AECII cells while concurrently preventing fibrosis through the inhibition of fibrocyte recruitment and proliferation, mediated by CXCL12 (134, 135). iv. The mechanism by which Tregs are recruited to skin and hair remains unclear (65). However, once present, Tregs curb the inflammatory actions of M1 macrophages and enhance wound healing and hair regeneration through the Jag1-Notch signaling pathway (136). v. Within skeletal muscle, IL-33 aids in the recruitment of Tregs to injury sites. These cells suppress inflammation mediated by M1 macrophages, thereby fostering a shift towards the resolution phase of healing. Tregs also directly stimulate satellite cell proliferation and differentiation through Areg. vi. In cardiac tissue, Tregs are drawn to the site via CCR5 signaling, involving chemokines like CCL3 and CCL4, which leads to the suppression of Th1 cell activities and inhibition of M1 macrophages (137).

### Metabolic regulation

The non-canonical function of Tregs in metabolic regulation highlights their importance in systemic homeostasis beyond mere immune modulation. Research has elucidated the involvement of Tregs in the modulation of lipid metabolism, insulin sensitivity, and overall energy balance (138, 139). These cells exert their effects in key metabolic sites, including adipose tissue, liver, and skeletal muscle, areas critical for metabolic homeostasis.

In adipose tissue, Tregs accumulate in visceral fat and contribute to the maintenance of insulin sensitivity and glucose homeostasis. This function is mediated by the secretion of anti-inflammatory cytokines such as IL-10 and TGF-β, which play roles in reducing inflammation — a known contributor to insulin resistance (140, 141). Moreover, adipose Tregs express the transcription factor PPAR-γ (peroxisome proliferator-activated receptor gamma), a master regulator of lipid metabolism, suggesting a direct role in lipid handling and storage (142, 143). Examination of the mechanisms underlying the function of Tregs in visceral adipose tissue (VAT) revealed that these cells, characterized by PPAR-γ expression, also express ST2 and depend on interleukin-33 (IL-33) for their accumulation in VAT (144–146). Administration of IL-33 to obese mice, which exhibit a reduced number of adipose Tregs, results in an expansion of these cells to levels seen in non-obese counterparts and leads to the restoration of insulin sensitivity (144). Although IL-33 signaling impacts other cell types such as innate lymphoid cells (ILCs), macrophages, and various additional cells, the critical role of ST2 signaling in sustaining visceral adipose tissue-Treg populations was confirmed through a Treg-specific knockout of the receptor (147, 148).

One present research identifies two unique Treg cell types, ST2+ and CXCR3+, which exhibit sex-specific distributions and functional differences influenced by hormonal variations (146). ST2+ Treg cells, found predominantly in males and driven by IL-33 and PPARγ, are essential for maintaining systemic glucose metabolism and controlling inflammation (149). Conversely, CXCR3+ Treg cells, more prevalent in females and influenced by IFN-γ and T-bet, play key roles in mitigating inflammation, especially under lean conditions (150). This study not only details the mechanisms through which these Treg cells regulate metabolic processes and inflammatory responses, including their interactions with other immune cells and response to environmental signals, but also emphasizes their potential as therapeutic targets in metabolic diseases like diabetes and obesity, highlighting the importance of understanding their regulatory mechanisms and developmental heterogeneity (146, 149).

Furthermore, Tregs influence liver metabolism, particularly in the context of inflammatory conditions such as non-alcoholic fatty liver disease (151, 152). By modulating the inflammatory milieu within the liver, Tregs can mitigate the progression of inflammation-driven hepatic damage, which is closely linked to metabolic dysregulation (153).

#### Support of the stem cell nich

Tregs exert profound effects on stem cell niches, impacting tissue regeneration and homeostasis through intricate mechanisms that are increasingly being elucidated in current research. The diverse roles of Tregs span several tissues, each characterized by unique interactions and signaling pathways that govern the fate and function of resident stem cells. Here, we try to explore the mechanisms by which Tregs influence stem cell niches in the bone marrow, skin, and intestines, highlighting the molecular underpinnings and potential therapeutic implications.

In the bone marrow, Tregs contribute to the maintenance of hematopoietic stem cell (HSC) quiescence and integrity through several pathways. Tregs express CXCR4, which is crucial for their homing to the bone marrow and positioning within the HSC niche (154). Once localized, Tregs modulate the HSCs via the secretion of adenosine, which is generated through the enzymatic activity of CD39 (2). This interaction is further exemplified by Treg-mediated inhibition of HSC proliferation and differentiation, a process reversed by antioxidant treatments that mitigate the oxidative milieu (155).

In skin, particularly around hair follicles, Tregs play a key role in modulating hair follicle stem cell (HFSC) cycles and regeneration (17). Tregs impact HFSC behavior through direct cell-cell contact and paracrine signaling mechanisms, notably involving the Notch pathway (156). Tregs increase the expression of the Notch ligand Jagged-1 (Jag-1), enhancing Notch signaling in HFSCs to promote hair growth and follicle regeneration (156). This process is critical during the telogen-to-anagen transition phase of the hair cycle, where Tregs have been shown to regulate the timing and extent of HFSC activation and subsequent hair follicle development (17).

In the intestinal mucosa, Tregs interact with intestinal stem cells through mechanisms involving MHC II (134, 157, 158). These interactions facilitate the maintenance of ISC integrity and function under inflammatory conditions, promoting tissue repair and barrier function. Tregs secrete IL-10, which not only dampens inflammatory responses but also supports the expansion and renewal of mesenchymal stromal cells that are crucial for ISC niche support (159). This regulatory axis is vital for preventing dysregulated immune responses from impairing ISC function, which is essential for maintaining intestinal homeostasis and responding to epithelial damage.

#### Regulation of angiogenesis

Tregs also exhibit capabilities in regulating angiogenesis, the process by which new blood vessels form from pre-existing vessels (160, 161). This non-canonical function of Tregs has significant implications for both tissue repair and tumor progression, highlighting their dual role in promoting or inhibiting angiogenesis under different pathological conditions.

Tregs could influence angiogenesis through several mechanisms, primarily involving the secretion of factors that can either promote or inhibit the angiogenic process. One of the key mediators in this context is vascular endothelial growth factor (VEGF), a potent angiogenic factor that Tregs can modulate indirectly through interactions with other immune cells or directly by cellular secretion (161, 162). By expressing or inducing the expression of VEGF, Tregs can enhance angiogenesis, facilitating tissue repair and regeneration (163). Conversely, Tregs can limit VEGF-induced angiogenesis through the secretion of anti-inflammatory cytokines such as TGF-β and IL-10, which are known to modulate inflammatory responses that influence angiogenesis (164, 165).

In addition, Tregs directly interact with endothelial cells, the primary cells involved in angiogenesis. Through direct cell-cell contact and the secretion of cytokines, Tregs can influence endothelial cell proliferation, migration, and tube formation (166, 167). These interactions are critical during the formation of new blood vessels, especially in contexts of wound healing and tissue regeneration.

**Figure 5:**
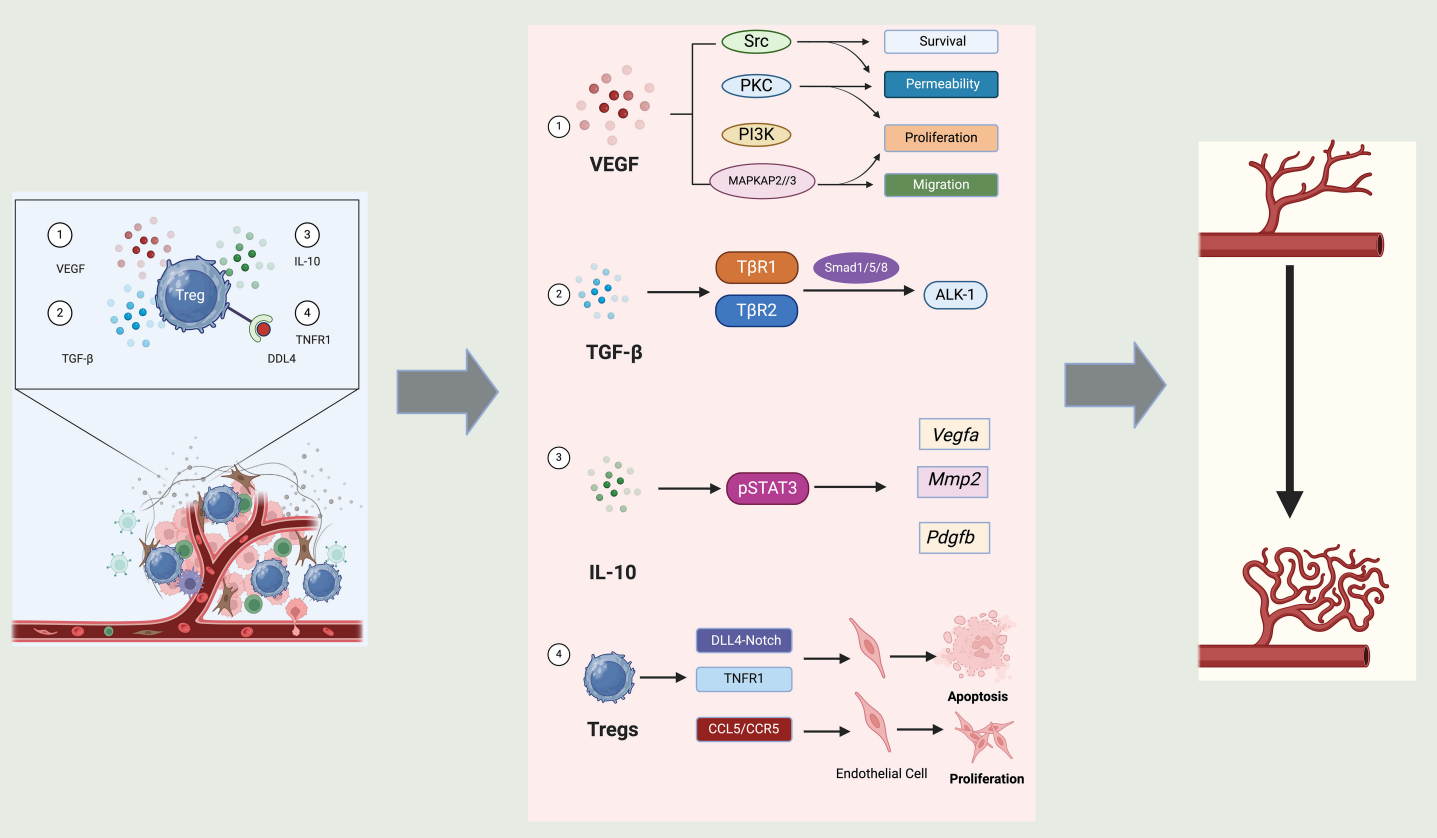
Role of Tregs in angiogenesis. Tregs influence angiogenesis through a variety of mechanisms mediated by distinct signals and interactions with endothelial cells. Firstly, Tregs contribute to the secretion of VEGF, a critical pro-angiogenic factor. This promotes several key functions in endothelial cells, including survival, permeability, proliferation, and migration, which are essential for new blood vessel formation. The signaling cascades involved, such as Src, PKC, PI3K, and MAPK pathways, underscore the multifaceted approach by which VEGF orchestrates the angiogenic process. Secondly, TGF-β secreted by Tregs interacts with its receptors, and through signaling intermediates like Smad1/5/8 and ALK-1, Tregs regulates the expression of angiogenic factors such as VEGF-A, MMP2, and PDGF-B. These molecules play crucial roles in modifying the extracellular matrix and promoting endothelial cell function, essential for angiogenesis. Thirdly, IL-10 produced by Tregs activates STAT3 signaling in target cells, which further influences the expression of angiogenic factors. This pathway underscores the anti-inflammatory and pro-angiogenic roles of IL-10, contributing to a conducive environment for vessel growth. Finally, Tregs directly interact with endothelial cells through mechanisms such as DLL4-Notch and TNFR1 signaling. These interactions can lead to endothelial cell apoptosis or proliferation, depending on the context, thereby influencing angiogenesis directly at the cellular level. Additionally, the interaction via CCL5/CCR5 pathways indicates a chemokine-mediated regulation of endothelial function by Tregs.

Moreover, Tregs modulate the function of other immune cells in the angiogenic process. For example, Tregs can inhibit the angiogenic contributions of macrophages and other T cell subsets by suppressing their pro-inflammatory and pro-angiogenic activities (168, 169). This regulation ensures that angiogenesis does not proceed unchecked, which is crucial in preventing pathological conditions such as tumor growth and metastasis. Tregs play a pivotal role in promoting angiogenesis through multiple mechanisms (160). They enhance vascular growth by secreting VEGF, which activates endothelial cell proliferation, migration, and survival via Src, PI3K, and MAPK pathways (161). Tregs also release TGF-β and IL-10, modulating angiogenic gene expression through Smad and STAT3 signaling, respectively. Furthermore, direct interactions with endothelial cells via DLL4-Notch, TNFR1, and chemokine pathways such as CCL5/CCR5 influence endothelial cell behavior (170). Collectively, these mechanisms highlight Tregs as active participants in shaping the vascular microenvironment across various physiological and pathological contexts (**Figure 5**).

#### Role of Tregs in angiogenesis

Tregs influence angiogenesis through a variety of mechanisms mediated by distinct signals and interactions with endothelial cells. Firstly, Tregs contribute to the secretion of VEGF, a critical pro-angiogenic factor. This promotes several key functions in endothelial cells, including survival, permeability, proliferation, and migration, which are essential for new blood vessel formation. The signaling cascades involved, such as Src, PKC, PI3K, and MAPK pathways, underscore the multifaceted approach by which VEGF orchestrates the angiogenic process (160, 171). Secondly, TGF-β secreted by Tregs interacts with its receptors, and through signaling intermediates like Smad1/5/8 and ALK-1, Tregs regulates the expression of angiogenic factors such as VEGF-A, MMP2, and PDGF-B. These molecules play crucial roles in modifying the extracellular matrix and promoting endothelial cell function, essential for angiogenesis (172). Thirdly, IL-10 produced by Tregs activates STAT3 signaling in target cells, which further influences the expression of angiogenic factors. This pathway underscores the anti-inflammatory and pro-angiogenic roles of IL-10, contributing to a conducive environment for vessel growth (173). Last but not least, Tregs directly interact with endothelial cells through mechanisms such as DLL4-Notch and TNFR1 signaling (174, 175). These interactions can lead to endothelial cell apoptosis or proliferation, depending on the context, thereby influencing angiogenesis directly at the cellular level. Additionally, the interaction via CCL5/CCR5 pathways indicates a chemokine-mediated regulation of endothelial function by Tregs (176).

Further research is needed to elucidate the detailed mechanisms by which Tregs regulate angiogenesis and to determine how these mechanisms vary among different tissues and disease states. Developing targeted therapies that manipulate Treg functions in angiogenesis offers promising prospects for the treatment of a wide range of diseases, necessitating a collaborative effort between immunology and vascular biology fields to explore these therapeutic potentials fully (177).

#### Engineering antigen-specific Tregs

**Figure 6:**
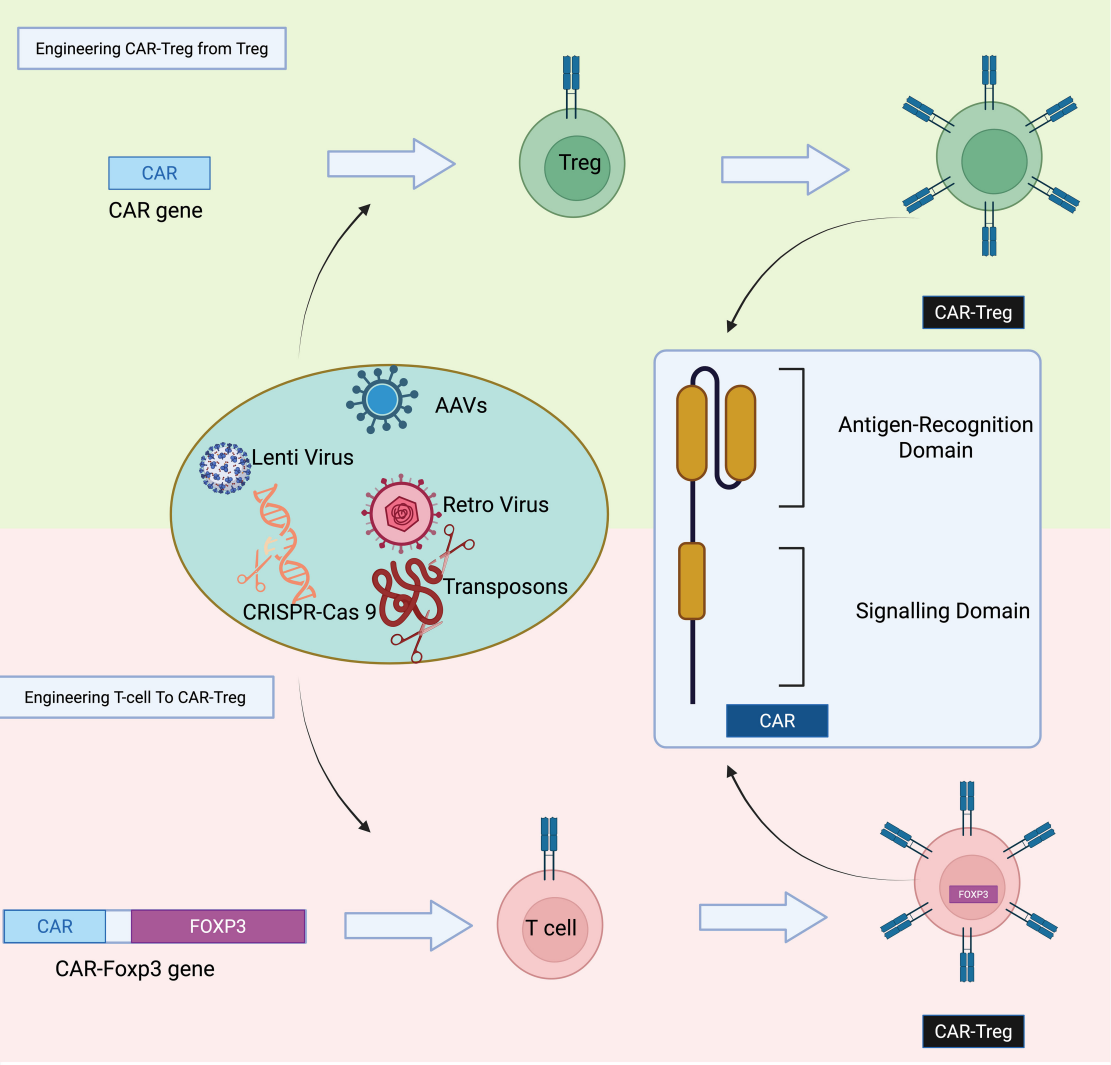
Strategies for generating CAR-Tregs. CAR-Tregs can be produced either by transducing polyclonal Tregs with a CAR construct or by co-transducing conventional T cells with both a CAR construct and the FoxP3 transcription factor gene to induce a regulatory phenotype.

Tregs engineered to express CARs, known as CAR Tregs, are generated through genetic modification techniques that introduce CAR constructs into T cells (**Figure 6** **) (** 178). This is typically achieved using viral vectors, such as lentiviral, retroviral, or adeno-associated viral systems, which facilitate stable integration of the CAR gene into the T cell genome. Alternatively, non-viral approaches like Sleeping Beauty or piggyBac transposon systems, and CRISPR-Cas9-based gene editing, offer site-specific integration with reduced risk of insertional mutagenesis (177, 179, 180). The starting cell population may include polyclonal Tregs, CD4⁺ T cells, or CD3⁺ T cells. In the case of polyclonal Tregs, cells are isolated and directly transduced with CAR constructs; however, their low abundance and potential phenotypic instability limit this approach. To overcome these limitations, CD4⁺ T cells can be co-transduced with CAR constructs and FOXP3 cDNA to induce a regulatory phenotype (181). This strategy enhances cell availability and helps maintain stable FOXP3 expression, which is essential for Treg function. These CAR Tregs are then expanded *ex vivo* under controlled conditions before being used for therapeutic applications, such as in autoimmunity, transplantation, or inflammation-related diseases. In 2022, a Phase 1/2 clinical trial (NCT04817774) investigated the safety and tolerability of autologous HLA-A2-specific CAR-Treg cells in kidney transplant recipients (182). A separate ongoing study (NCT05234190) is set to evaluate similar CAR-Treg therapy in liver transplant patients (182). These trials underscore the growing momentum and scientific interest in advancing CAR-Treg–based immunotherapies within the field of transplantation.

#### Strategies for generating CAR-Tregs

CAR-Tregs can be produced either by transducing polyclonal Tregs with a CAR construct or by co-transducing conventional T cells with both a CAR construct and the FoxP3 transcription factor gene to induce a regulatory phenotype.

Introducing recombinant TCRs into Tregs enables antigen-specific activation by recognizing peptides presented by MHC molecules on antigen-presenting cells (182). Preclinical studies have shown that TCR-engineered Tregs specific for allo-MHC class II can prolong graft survival in transplant models, and autoreactive TCR-Tregs have shown therapeutic efficacy in autoimmune disease models such as EAE and type 1 diabetes (182–184). Future strategies may involve using TCRs naturally derived from tissue-resident Tregs to enhance tissue-specific immunoregulation, though knowledge about these TCRs and their target peptides remains limited.

To overcome the challenge of limited knowledge about relevant antigens and to enable MHC-independent activation, Treg cells have recently been engineered with artificial immune receptors (AIRs)—biosensors that detect inflammatory cues instead of antigens (185). These AIRs are designed to recognize membrane-bound inflammatory ligands such as TNF-α, TL1A (Tumor Necrosis Factor Superfamily Member 15), and LIGHT (Tumor Necrosis Factor Superfamily Member 14) (185). Structurally like CARs, AIRs include CD3ζ and CD28 intracellular signaling domains, enabling Tregs to respond to local inflammation. RNA sequencing has shown that AIR stimulation induces TCR-like signaling and promotes Treg proliferation. Importantly, AIRs respond only to membrane-bound ligands, enhancing tissue specificity. *In vivo*, AIR-Tregs expressing lymphotoxin-β receptor (LTBR) demonstrated superior protection against GvHD compared to polyclonal Tregs (185).

Though off-target effects are a potential concern, safety switches like inducible caspase 9 can be integrated to eliminate AIR-Tregs if needed. Comparative studies among TCR-Tregs, CAR-Tregs, and AIR-Tregs are needed to assess which approach offers better long-term persistence and therapeutic efficacy in chronic inflammatory settings or transplantation.

### Tregs involved in the treatment of non-cancerous diseases

Tregs present unique opportunities for therapeutic innovation aimed at addressing the complex pathologies underlying many autoimmune and inflammatory diseases. Unlike other treatment modalities, Treg therapies have demonstrated the capacity to induce immune tolerance, allowing for short-term treatments that lead to prolonged periods of remission. Additionally, Tregs can function effectively in both lymphoid and non-lymphoid tissues, utilizing diverse mechanisms to modulate immune responses (186). In various preclinical studies, Treg-based adoptive immunotherapy has successfully reduced systemic inflammation and targeted organ damage across multiple autoimmune conditions such as inflammatory bowel disease (187), type 1 diabetes (188), experimental autoimmune encephalomyelitis (189), collagen-induced arthritis (190), and in models of organ transplant rejection and non-immune diseases like stroke (191) and amyotrophic lateral sclerosis (192), where inflammation contributes to the pathology. For example, researchers have recently developed a novel strategy by engineering Tregs to express a chimeric antigen receptor targeting the interleukin-23 receptor, thereby generating IL-23R CAR-Tregs as a potential therapy for Crohn’s disease (193). Given the critical role of IL-23R signaling and IL-23R-expressing mucosal cells in the development of ﻿inflammatory bowel diseases, targeting IL-23R using CAR-T cells represents a promising therapeutic strategy. The study showed that IL-23R-CAR-Treg cells displayed CAR-dependent suppressive effects on target cells *in vitro* and effectively protected mice from induced colitis (194–196). Following this, other research teams utilized autologous polyclonal Treg cells that were expanded ex vivo as a form of cellular therapy for treating inflammatory bowel diseases, as explored in clinical trials (NCT04691232; NCT03185000). A recent phase I, fast-track dose-escalation clinical trial in ulcerative colitis showed that a single infusion of Treg cells led to clinical improvement in certain subgroups of patients with active disease (68).

However, the ability of Tregs to act on nearby cells and the pivotal role of tissue-specific Tregs in managing local autoimmunity suggest that Tregs with single-antigen specificities may offer enhanced efficacy and safety. Studies in autoimmune and transplant settings have shown that Tregs expressing a singular T cell receptor can suppress autoreactive Teff cells and foster long-term tolerance. Early human trials indicate that alloantigen-specific Treg cells are promising in transplantation (197, 198). Recent advancements in synthetic biology have further enhanced Treg specificity through the introduction of specific TCRs and CARs, targeting inflamed tissues directly (199, 200). These innovations have not only proven effective in preclinical models but also demonstrated a significant increase in activity compared to polyclonal Treg populations. Therefore, with their versatile functionality across different diseases and the potential for genetic engineering, Treg therapies represent a groundbreaking approach to developing profound and enduring treatments.

### Tregs involved in the treatment of cancer therapeutics

Recent advances in understanding and manipulating Tregs are transforming the landscape of cancer therapeutics. Tregs have a paradoxical role in cancer therapeutics by often enhancing tumor survival through immune suppression. This has led to a concentrated effort to either selectively deplete or functionally modulate Tregs in the tumor microenvironment to improve cancer immunotherapy outcomes without disrupting their protective role in healthy tissues (2, 201).

One significant area of development involves targeting Tregs that infiltrate tumors (TI-Tregs). These cells express high levels of immune checkpoint molecules such as CTLA-4 and PD-1, which are also targets of widely used cancer immunotherapies like ICB antibodies (147, 202). Research has shown that while blocking these pathways can reactivate effector T cells, it can also inadvertently enhance Treg suppression, underscoring the need for approaches that specifically target TI-Tregs without affecting those in non-tumoral tissues (203).

Recent studies have focused on exploiting the unique expression profiles of surface and intracellular molecules on TI-Tregs. For instance, differences in cytokine and metabolite dependency between TI-Tregs and conventional T cells provide opportunities for selective targeting (204–207). Molecules such as CD25 and CCR8 have emerged as potential targets to deplete TI-Tregs while sparing those necessary for immune homeostasis in healthy organs (208, 209). These approaches aim to evoke potent anti-tumor immunity while minimizing the risk of autoimmune responses.

The development of next-generation cell therapies, including engineered Tregs with CARs or modified TCRs, is also progressing (210–213). These engineered Tregs are designed to accurately target tumor cells or their microenvironment, enhancing specificity and minimizing off-target effects. Advanced gene-editing techniques, such as CRISPR/Cas9, are used to enhance the stability and functional properties of these cells under the harsh conditions of the tumor microenvironment (214, 215).

Furthermore, combination therapies that integrate Treg modulation with traditional cancer treatments are being tested. For example, combining Treg depletion strategies with immune checkpoint blockades or conventional chemotherapy has shown promise in preclinical models, suggesting that dual targeting of immune suppression and tumor cells can synergistically improve treatment outcomes (216–218).

### Microbiota–Treg crosstalk in cancer and tissue repair

Unlike thymus-derived “natural” Tregs that form in a largely microbe-independent manner, RORγt⁺ Tregs in the colon are peripherally induced by commensal microbiota and carry a distinct microbial imprint (219–221). Gut bacteria produce metabolites—such as short-chain fatty acids (SCFAs) from dietary fiber and tryptophan catabolites—that drive the differentiation of these Foxp3⁺RORγt⁺ Tregs from naïve CD4⁺ T cells (222). SCFAs such as notably butyrate and propionate enhance Foxp3 expression through epigenetic modulation, thereby expanding the colonic Treg pool (223). Likewise, microbial tryptophan metabolites (e.g. indoles) serve as ligands for the aryl hydrocarbon receptor (AhR) on T cells, promoting the development of IL-10–producing RORγt⁺ Tregs. In parallel, commensal signals induce local dendritic cells and RORγt⁺ antigen-presenting cells to favor TGF-β–dependent Treg induction (224). The result is a specialized colonic Treg subset that co-expresses RORγt and is highly adapted to the intestinal environment – they often express gut-homing receptors (like CCR9) and secrete IL-10, which reinforces immune tolerance to microbiota (219, 225). By contrast, thymic Tregs recognize self-antigens and can function without microbial input, highlighting a fundamental difference in ontogeny and function between these subsets. Importantly, the influence of microbiota on RORγt⁺ Tregs extends beyond the gut, affecting systemic immunity, tissue repair, and cancer (13, 226–228). Colonic RORγt⁺ Tregs have been shown to migrate to distant sites of injury: for example, after muscle damage they transiently accumulate in the muscle to restrain IL-17A–mediated inflammation and thereby promote stem cell–driven regeneration (13). This “emissary” role suggests that a healthy microbiota can pre-condition regulatory T cells that aid in the resolution of inflammation and tissue healing throughout the body (13). In the context of cancer, microbiota-Treg interactions become a double-edged sword (229). On one hand, robust induction of RORγt⁺ Tregs by commensals helps keep chronic tissue inflammation in check – a factor known to reduce inflammation-driven tumorigenesis (229). On the other hand, an abundance of microbiota-educated Tregs in the tumor microenvironment may suppress anti-tumor effector responses, allowing tumors to evade immune surveillance (230). Thus, the balance of microbially tuned tolerance versus immunity can shape cancer outcomes. Understanding this crosstalk opens avenues for therapy: manipulating gut microbiota or their metabolites might modulate Treg activity to either enhance immune-mediated tumor clearance or, conversely, to reinforce regulatory pathways that limit tissue-damaging inflammation. In summary, RORγt⁺ Tregs represent a critical nexus between the microbiome and the immune system, influencing inflammatory diseases, cancer immunity, and the capacity for tissue regeneration.

### Challenges and key features to be addressed in developing Treg therapeutics

Developing therapeutic strategies based on Tregs presents a promising frontier in cancer immunotherapy, yet it confronts numerous challenges and intricacies that necessitate detailed consideration. The key to advancing Treg-based therapies lies in overcoming these hurdles while harnessing the unique properties of Tregs to effectively target and modulate the tumor microenvironment.

i. Antigen-specificity: The antigen specificity of Tregs within the TME has garnered significant attention in recent years. While Tregs can suppress immune responses through antigen-independent mechanisms such as IL-2 consumption, secretion of inhibitory cytokines (e.g., IL-10, TGF-β), and CTLA-4-mediated modulation of dendritic cells, increasing evidence suggests that antigen recognition plays a crucial role in their tumor-suppressive function (231). Tumor-infiltrating Tregs often display a restricted TCR repertoire, indicating clonal expansion in response to tumor antigens. Several preclinical studies support this concept: in murine models, Tregs specific for tumor-associated antigens such as gp100 or carcino-embryonic antigen were shown to suppress effective antitumor T cell responses more potently than polyclonal Tregs (231–233). Furthermore, engineered Tregs expressing tumor-specific TCRs or CARs exhibit enhanced immunosuppressive activity within tumors (211). These findings imply that antigen specificity not only guides the accumulation of Tregs in tumors but may also enhance their suppressive capacity. However, the extent to which TCR engagement is required for Treg-mediated suppression remains debated, and further research is needed to dissect the balance between antigen-dependent and -independent mechanisms. Understanding this balance is critical for developing targeted strategies to modulate Tregs in cancer immunotherapy.

ii. Stability and Persistence in the Tumor Microenvironment: The suppressive and hypoxic conditions within tumors can affect the survival, function, and stability of infused or endogenously expanded Tregs. Modifying Tregs to enhance their resilience and functional stability in such hostile environments is critical (234, 235). This might involve genetic or pharmacological modulation of pathways involved in Treg survival and metabolic fitness, such as enhancing their oxidative phosphorylation capacity or their ability to respond to stress signals within the tumor microenvironment (236).

iii. Balancing Suppression: While the immunosuppressive functions of Tregs are beneficial in preventing autoimmunity and controlling inflammation, in the context of cancer, they can hinder effective anti-tumor immune responses. Innovatively designed strategies that modulate the suppressive functions of Tregs—potentially through transient and reversible approaches—are essential to maintain the balance between suppressing autoimmunity and enabling robust anti-tumor immunity.

iv. Off-Target Effects and Safety Concerns: The systemic effects of enhancing or inhibiting Tregs can lead to unintended consequences, such as increased susceptibility to infections or the development of autoimmune disorders. Developing localized delivery systems or ensuring that Treg activity is confined to the tumor site can mitigate these risks. Additionally, the potential for Tregs to convert into effector T cells under certain inflammatory conditions needs careful consideration to prevent adverse outcomes (11, 213).

v. Integration with Existing Therapies: Combining Treg-based approaches with existing cancer therapies such as chemotherapy, radiotherapy, and other immunotherapies could enhance overall treatment efficacy (237–239). However, the interactions between these therapies and Treg functions are complex and not fully understood. Research into how Tregs interact with other therapeutic agents and how they can be combined synergistically to improve patient outcomes is vital (11).

vi. Monitoring and Measuring Success: Effective biomarkers that can accurately monitor the function and impact of Treg therapies in real-time are lacking. Developing such biomarkers would enable better tracking of therapy efficacy and adjustment of treatment protocols in a personalized manner.

In conclusion, while the therapeutic potential of Tregs in cancer treatment is substantial, realizing this potential requires overcoming significant scientific and clinical challenges. Addressing these issues through innovative research and clinical trials will be crucial for the successful integration of Treg therapies into standard cancer treatment regimens. The journey from concept to clinic, therefore, involves not only understanding the fundamental biology of Tregs but also developing new technologies and therapeutic paradigms that can safely and effectively exploit these cells in the fight against cancer.

## Concluding remarks

Tregs have emerged as central players not only in maintaining immune tolerance but also in orchestrating a wide range of physiological processes with significant therapeutic implications. The evolving understanding of Treg biology highlights their multifaceted roles across various disease contexts, including cancer, autoimmune disorders, metabolic diseases, and regenerative medicine.

In regenerative medicine, Tregs demonstrate significant potential through their ability to modulate inflammation and support tissue repair. Enhancing Treg function or mimicking their reparative actions offers promising strategies for improving outcomes in conditions characterized by chronic inflammation and impaired healing. Furthermore, Tregs contribute to angiogenesis and the maintenance of stem cell niches, suggesting a broader role in facilitating tissue regeneration. These insights emphasize the need to further dissect the molecular mechanisms by which Tregs interact with regenerative pathways, particularly in inflammatory and degenerative disease models.

Beyond tissue repair, Tregs are increasingly recognized for their regulatory influence over metabolic pathways. Their ability to modulate metabolic homeostasis positions them as attractive targets for treating complex metabolic disorders such as diabetes and metabolic syndrome. Therapeutically manipulating Tregs in this context could represent a novel immune-based approach to restoring metabolic balance and reducing chronic systemic inflammation.

In cancer, the dualistic role of Tregs presents both challenges and opportunities. While they are essential for maintaining immune tolerance, Tregs also suppress anti-tumor immunity within the tumor microenvironment, facilitating immune evasion and tumor progression. Therapeutic strategies that selectively disrupt the immunosuppressive functions of intratumoral Tregs—such as CAR-Tregs, monoclonal antibodies, and small-molecule inhibitors—have shown promise in preclinical and early clinical studies. These approaches aim to shift the immune balance toward effective tumor eradication while preserving systemic immune regulation.

Despite these advancements, several key challenges remain. Ensuring the stability and functional specificity of engineered or expanded Tregs in hostile environments, such as inflamed or hypoxic tumor tissues, is critical. Genetic modifications that enhance Treg resilience under such conditions are being explored. Equally important is the development of targeted delivery systems and reliable biomarkers to monitor Treg activity and therapeutic efficacy in real-time, minimizing off-target effects and improving patient stratification.

Taken together, the therapeutic manipulation of Tregs offers a compelling avenue for treating a spectrum of diseases. Their diverse functions—from immune suppression to tissue regeneration and metabolic regulation—underscore their potential as powerful tools in modern medicine. Future research should focus on refining Treg-based therapies to enhance their specificity, stability, and context-dependent functionality. By integrating advances in immunology, genetic engineering, and biomarker discovery, we can unlock the full therapeutic potential of Tregs and develop next-generation treatments that are both effective and safe across a wide range of clinical settings.
